# Differential diagnosis of benign and malignant vertebral compression fractures based on CT radiomics model

**DOI:** 10.3389/fonc.2025.1697550

**Published:** 2026-01-02

**Authors:** Xinrui Liu, Song Chen, Yifan Wang, Jiashi Cao, Zhuangfei Niu, Yuxian Jin, Xingdan Pan, Zhengwei Zhang, Tielong Liu, Wei Liang, Panfeng Yu, Weiwei Zou

**Affiliations:** 1 School of Health Science and Engineering, University of Shanghai for Science and Technology, Shanghai, China; 2 Shanghai Changzheng Hospital Department of Radiology, Shanghai, China; 3 Shanghai Baoshan District Wusong Central Hospital (Zhongshan Hospital Wusong Branch, Fudan University), Shanghai, China; 4 Navy Medical Center, The Navy Medical University, Shanghai, China; 5 Shanghai Changzheng Hospital Department of Orthopedic Oncology, Shanghai, China; 6 Department of Pathology, Shanghai Changzheng Hospital, Shanghai, China; 7 Shanghai 411 Hospital, Affiliated Hospital of Shanghai University, Shanghai, China; 8 Department of Orthopedic Oncology, Peking University People’s Hospital, Beijing, China

**Keywords:** clinical predictors, computed tomography (CT), machine learning, radiomics, vertebral compression fractures (VCF)

## Abstract

**Objectives:**

This study aims to develop a CT radiomics-based predictive model integrating clinical characteristics to distinguish benign and malignant vertebral compression fractures (VCFs).

**Methods:**

We retrospectively analyzed 208 patients with VCFs treated at our institution between January 2020 and November 2024. Patients were randomly divided into a training cohort (n = 145) and a validation cohort (n = 63). CT images were obtained, and three-dimensional lesion regions were manually segmented. A total of 1,316 radiomics features were extracted. Dimensionality reduction was performed using least absolute shrinkage and selection operator (LASSO) regression analysis and 5-fold cross-validation to identify key features. Univariate and multivariate analyses were used for identifying independent clinical predictors. Three models were constructed: a clinical model, a radiomics model, and a combined clinical-radiomics model. Model performance was evaluated using area under the receiver operating characteristic (ROC) curve (AUC), accuracy (ACC), sensitivity (SEN), specificity (SPE), positive predictive value (PPV), and negative predictive value (NPV). Predictive efficacy and clinical utility were further assessed via ROC curves, calibration plots, and decision curve analysis (DCA), along with clinical impact curves (CIC) and net reduction curves. The Delong test was used for statistical comparisons among different models, and a nomogram was developed to facilitate the visualization of the optimal model.

**Results:**

Carbohydrate antigen 125 (CA125) and posterior vertebral involvement were identified as independent clinical predictors. The combined model achieved the highest AUC value of 0.846 in the validation cohort, followed by the radiomics model (0.842), and the clinical model (0.640). Calibration curves and DCA confirmed its superior predictive accuracy and clinical benefit.

**Conclusions:**

The CT-based clinical-radiomics model demonstrated robust performance in differentiating benign from malignant VCFs and holds promise for guiding individualized patient management.

## Introduction

Vertebral compression fractures (VCFs) are prevalent spinal disorders, characterized by the collapse of vertebral body height. Annually, over 1.4 million new cases are reported worldwide, with an increasing incidence attributed to the aging global population (1, 2). VCFs predominantly occur at the thoracolumbar spine and typically present with symptoms such as back pain, kyphotic deformity, and functional impairment. Severe cases may be accompanied by spinal cord injury or paralysis, significantly diminishing patients’ quality of life, contributing to psychological distress, and increasing the social and economic burden (3, 4). VCFs are categorized into benign and malignant types based on their etiology. Benign VCFs often originate from conditions such as osteoporosis, trauma, benign lesions such as hemangiomas, with osteoporotic VCFs (OVCFs) being particularly prevalent among postmenopausal women and elderly men (5). Malignant VCFs primarily result from primary or metastatic malignances of the spine (6). The spine is the third most common site for cancer metastases, accounting for 10% to 15% of all metastatic cases (5, 7). Due to the substantial differences existing in the treatment strategies and prognostic outcomes between benign and malignant VCFs, early and precise differential diagnosis is essential for formulating individualized treatments.

Imaging examination plays an essential role in the diagnosis and differential diagnosis of VCFs. Magnetic resonance imaging (MRI) is regarded as an essential modality for diagnosing malignant VCFs due to its high sensitivity to bone marrow abnormalities. Typical MRI findings include paravertebral soft tissue masses, pedicle destruction, and posterior vertebral body bulging (8). However, similar signs could be also observed in benign VCFs that have paraspinal or epidural blood with edema (9). In addition, MRI is limited by high cost, long acquisition times, and contraindications in certain patients, restricting its routine clinical use. Computed tomography (CT) has the advantages of rapid scanning, high spatial resolution, and superior visualization of bony structures, which is widely employed for assessing fracture morphology and cortical bone integrity (10). Although CT offers inferior soft tissue contrast compared to MRI, its widespread availability and practicality make it especially valuable for initial diagnosis and in primary care settings (11). Notably, CT is often insufficient to diagnose VCFs, especially in patients without the history of significant trauma or malignancy. The situation may get even worse when there has no suitable condition to perform biopsy (12, 13). Traditional diagnosis relies heavily on the clinician’s experience, and the absence of typical imaging or sufficient clinical data significantly increases the risk of misdiagnosis or missed diagnosis. Thus, there is an urgent need to develop objective, quantitative, and reproducible tools to aid the differential diagnosis between benign and malignant VCFs.

Radiomics offers a promising approach to address this challenge. This technique allows comprehensive characterization of lesions beyond human visual assessment by extracting a large number of quantitative features from medical images, such as texture, shape, and intensity (14, 15). It has been implemented in clinical-decision support systems to enhance the efficacy of diagnosis, prognosis prediction, disease staging, and treatment response evaluation (16). Several studies have explored the application of radiomics for VCF diagnosis with promising results through MRI-based models (17–21). In contrast, research in CT-based radiomics is currently limited; however, it offers significant potential for widespread clinical application owing to the accessibility and efficiency of CT imaging.

We proposed the hypothesis that radiomic features derived from CT could effectively differentiate between benign and malignant VCFs. The objective of this study was to develop predictive models that integrate CT radiomic features with clinical parameters in patients with VCFs. By systematically evaluating the diagnostic performance of these models, we aim to establish an evidence-based decision-support framework to facilitate personalized management of VCFs.

## Materials and methods

### Patient datasets

**Figure 1:**
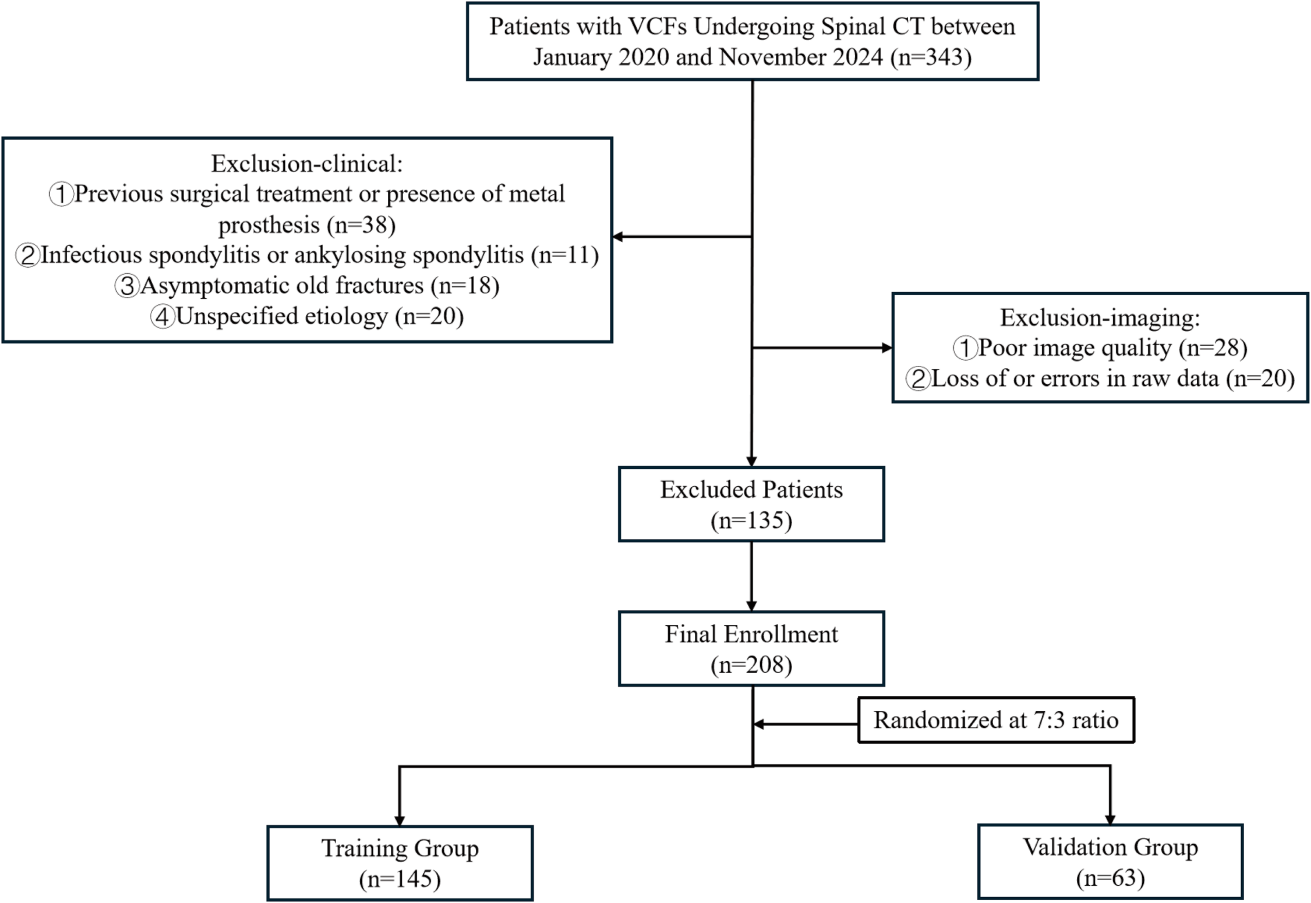
Patient screening flowchart. *VCFs*, vertebral compression fractures.

**Figure 2:**
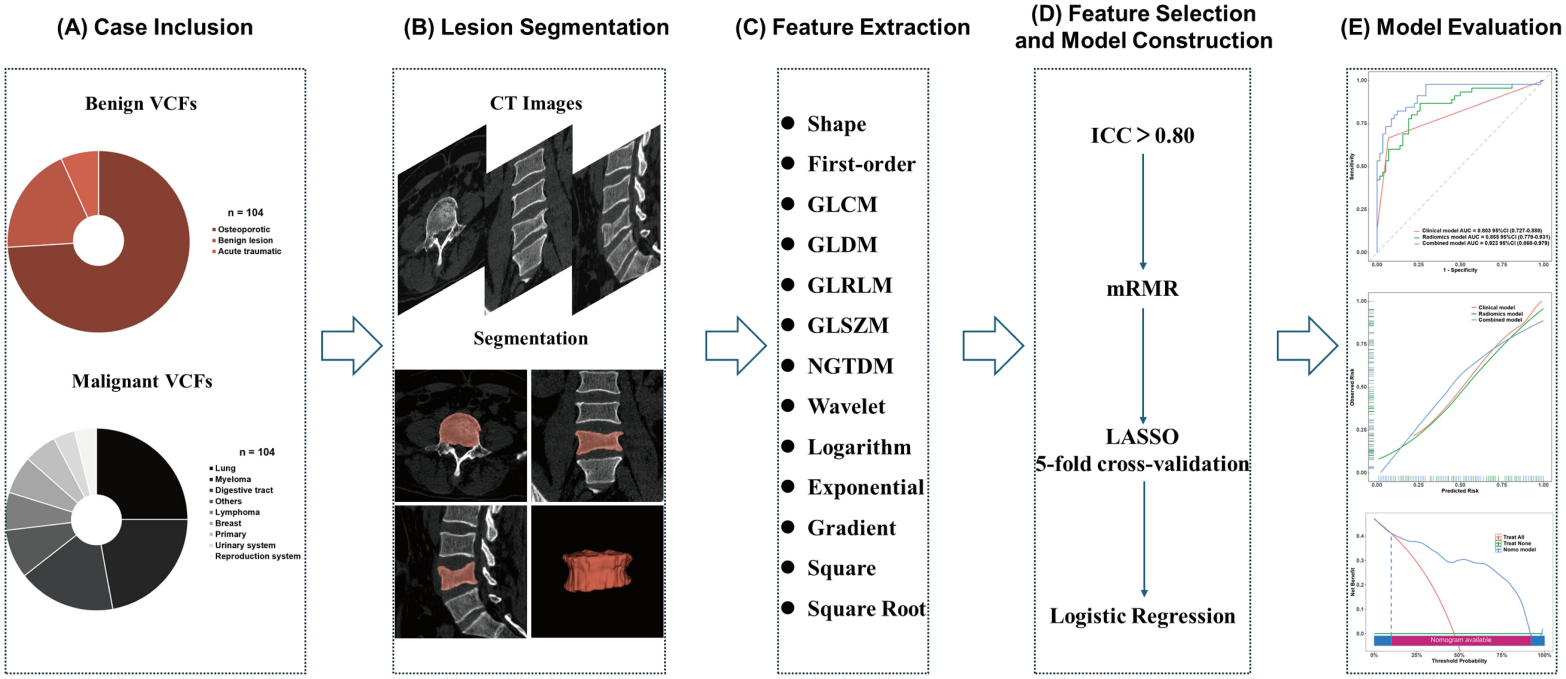
The radiomics workflow. **(A)** Case Inclusion: Distribution of enrolled benign and malignant VCFs. **(B)** Lesion Segmentation: Process of segmenting VCFs lesions from CT images. **(C)** Feature Extraction: Extraction of multi-type features from segmented lesions. **(D)** Feature Selection and Model Construction: Pipeline of feature screening, validation, and logistic regression model building. **(E)** Model Evaluation: Performance assessment of the classification model via curve visualization. CT, computed tomography; GLCM, gray level co-occurrence matrix; GLDM, gray level dependence matrix; GLRLM, gray level run length matrix; GLSZM, gray level size zone matrix; NGTDM, neighboring gray tone difference matrix; ICC, intraclass correlation coefficient; mRMR, minimum redundancy maximum relevance; LASSO, least absolute shrinkage and selection operator.

This study was approved by the Institutional Review Board of our center, with a waiver of informed consent granted due to its retrospective design. Patients diagnosed with VCFs were enrolled in this study who underwent spinal CT scans between January 2020 and November 2024. For the purpose of this study, VCFs were classified into two subtypes. Benign VCFs resulting from non-neoplastic causes, including osteoporotic, acute traumatic, or benign lesions like hemangioma; fresh osteoporotic fractures had acute onset or marrow edema on MRI (if available), chronic ones lacked edema with chronic documentation (22). Malignant vertebral involvement by primary or metastatic neoplasm, confirmed by histopathology or multidisciplinary diagnosis integrating imaging progression and clinical course (7). Clinical records and laboratory findings were concurrently retrieved. The inclusion and exclusion criteria were developed in consultation with a multidisciplinary team comprising orthopedic surgeons, radiologists, and pathologists. All disagreements were discussed until a consensus was reached. The inclusion criteria were as follows: (1) patients were initially confirmed as VCFs by imaging examination and pathological diagnosis for those with spinal tumors; (2) had complete imaging, clinical, and pathological (malignant VCFs) information. The exclusion criteria were detailed below: (1) patients had prior surgical intervention or metallic implants; (2) infectious spondylitis or ankylosing spondylitis; (3) asymptomatic chronic fractures; (4) suboptimal image quality or incomplete raw data; (5) undetermined fracture etiology. The final cohort comprised 208 patients with 377 affected vertebral bodies, randomly allocated into training (n = 145) and validation (n = 63) cohorts at a 7:3 ratio (**Figure 1**). Patient-level splitting was applied to ensure that all vertebrae from the same patient were assigned to only one cohort. The distribution of cases of VCFs is summarized in **Figure 2A**. The malignant VCFs section shows the proportion of primary spinal tumor and spinal metastatic tumors. Other tumors include thyroid cancer, nasopharyngeal cancer, adenoid cystic carcinoma, etc.

### CT scanning

CT imaging was performed using Philips Brilliance iCT (256-detector row), Philips Ingenuity (128-detector row), Siemens Somatom Force (dual-source), and United Imaging uCT 760 (320-detector row) scanners. Scan coverage encompassed the lesion site with inclusion of at least two adjacent vertebral segments above and below the affected vertebrae. All scans adhered to standardized parameters: 120 kV tube voltage, automated tube current modulation (80–400 mA), noise index of 18-20 HU, detector collimation of 0.6-0.625 mm, rotation time of 0.33-0.5 s, and pitch factor of 0.8-1.0. Reconstructed images maintained consistent parameters across protocols, including 1.0 mm slice thickness, 0.5-0.7 mm reconstruction interval, 512 × 512 matrix, and bone algorithm convolution kernels. Postprocessing standardization employed uniform bone window settings (window width 1500 HU; level 400 HU) across all scanner platforms to ensure cross-device consistency in visualizing osseous microarchitecture.

### Image segmentation and radiomics feature extraction

The radiomics workflow comprises five steps: (1) case inclusion, (2) lesion segmentation, (3) feature extraction, (4) feature selection and model construction, and (5) model evaluation (**Figure 2**). Lesion segmentation was performed by two experienced radiologists with 10-year and 5-year clinical experience, respectively. Both of them were blinded to the histopathological, clinical information, and group assignment of all patients. In cases of segmentation disagreement, consensus was achieved through joint review and discussion of the imaging data. Regions of interest (ROIs) were manually outlined using 3D Slicer software (version 5.7.0) on 1-mm sagittal CT slices to cover the entire affected vertebrae, including cortical margins and bilateral pedicle anterior walls, while excluding nearby intervertebral discs and fat tissue. For areas with cortical disruption, virtual continuity lines were aligned with the opposite intact cortices to maintain ROI integrity. Feature extraction was performed using PyRadiomics-3.0 on preprocessed DICOM data, producing multidimensional features like shape, first-order, and wavelet. To evaluate feature reproducibility, 30 random CT datasets were initially segmented by radiologist A. An independent re-segmentation was subsequently performed by radiologist B. Features with an intraclass correlation coefficient (ICC) above 0.80 were retained for further analysis.

### Feature selection and establishment of the radiomics model

A total of 1,316 radiomics features were initially extracted from CT plain scanning images. After evaluating feature reproducibility using the ICC, 874 features with ICC values greater than 0.80 were retained for further analysis. A two-step dimensionality reduction process was then applied. First, the minimum redundancy maximum relevance (mRMR) algorithm was used to perform feature selection based on maximum redundancy and minimum correlation. Second, least absolute shrinkage and selection operator (LASSO) regression with 5-fold cross-validation was employed to determine the optimal penalty parameter (λ), selecting features with nonzero coefficients to construct the radiomics signature. A logistic regression-based machine learning model was developed with all features subjected to Z-score normalization before model training. 5-fold cross-validation was utilized for hyperparameters optimization. The optimal hyperparameter combination was identified on the training set, and this model was preserved as a candidate radiomics predictor.

### Establishment of the clinical model and combined model

Clinical parameters were first analyzed using univariate method. Continuous variables were assessed with either the independent t-tests or Mann-Whitney U tests, depending on data distribution, while categorical variables were evaluated using chi-square test. Variables with *p* values less than 0.05 were selected for subsequent multivariate analysis. Stepwise logistic regression (threshold *p* < 0.05) was applied to identify independent predictors, which were subsequently used to construct the clinical model. The combined model was developed by linearly fusing the radiomics score (Rad-score) with clinical predictors, and the fusion coefficients were optimized via 5-fold cross-validation.

### Performance evaluation

Model performance was comprehensively assessed using receiver operating characteristic (ROC) curve analysis. The Delong test was used to compare area under the curve (AUC) values among the three models (a radiomics model, a clinical model and a combined model). Classification performance metrics were calculated for both the training and validation cohorts, including accuracy (ACC), sensitivity (SEN), specificity (SPE), positive predictive value (PPV), and negative predictive value (NPV). Calibration curves were used to assess the agreement between predicted probabilities and actual outcomes. To facilitate the visualization of the integrated prediction algorithm, a nomogram was constructed. Finally, decision curve analysis (DCA) was conducted to measure the clinical net benefit of model across a range of threshold probabilities, thereby evaluating clinical utility. Clinical impact curves (CIC) illustrate the clinical impact by showing the number of high-risk patients identified and true events at different thresholds. Net reduction curves quantify the avoidance of unnecessary interventions per patient across various thresholds, further evaluating model’s utility in optimizing clinical resource.

### Statistical analysis

All analyses were performed using R software (version 4.2.1, http://www.Rproject.org). For quantitative data, normally distributed variables were presented as mean ± standard deviation (SD), while non-normally distributed variables were shown as median (interquartile range, Q1-Q3). Categorical variables were described using frequencies (n) and percentages (%). Intergroup comparisons were conducted using independent t-tests for normally distributed continuous variables, Mann-Whitney U tests for non-normally distributed continuous variables, and chi-square tests for categorical variables. For all reported statistical associations, including AUC values and odds ratios (OR), 95% confidence intervals (95% CI) were calculated to quantify estimation precision. Feature selection and model construction utilized the “mRMRe” package to implement mRMR algorithms, identifying features strongly associated with clinical outcomes. Variable compression and selection were achieved through LASSO regression via the “glmnet” package, followed by multivariable logistic regression analysis using the “glm” package to develop predictive models. Visualization and evaluation components included nomograms, calibration curves, and DCA generated by the “rms” package, ROC curves plotted with “ggROC” package, and multi-model ROC comparisons performed using the “ROCR” package. All statistical tests were two-tailed, with *p* < 0.05 considered statistically significant.

## Results

### Baseline characteristics

A total of 208 patients with VCFs were randomly allocated to a training cohort (n = 145, 70%; 77 benign and 68 malignant cases) and a validation cohort (n = 63, 30%; 27 benign and 36 malignant cases) at a 7:3 ratio. **Table 1** summarizes the baseline characteristics of both cohorts. Within the training cohort, a statistical difference was observed in vertebral segment distribution between benign and malignant cases (*p* = 0.001). Additionally, no statistically significant differences were observed between the training and validation cohorts regarding age, sex, or fracture quantity, indicating comparable baseline distributions.

**Table 1:** Baseline characteristics of benign and malignant vertebral compression fractures.

Baseline characteristics	Training (n=145)		Independent t-tests	Validation (n=63)		Independent t-tests
	Begin (n=77)	Malignant (n=68)	*p* value	Begin (n=27)	Malignant (n=36)	*p* value
Age at diagnosis	64.38±12.29	61.60±12.98	0.190	65.00±11.45	62.22±12.63	0.366
Sex			0.075			0.135
Female	54 (70.13)	37 (54.41)		18 (66.67)	16 (44.44)	
Male	23 (29.87)	31 (45.59)		9 (33.33)	20 (55.56)	
Segmental distribution			0.001			0.052
Cervical vertebra (C1-7)	1 (1.30)	5 (7.35)		0 (0.00)	3 (8.33)	
Upper thoracic vertebra (T1-4)	0 (0.00)	7 (10.29)		1 (3.70)	5 (13.89)	
Middle thoracic vertebra (T5-8)	4 (5.19)	6 (8.82)		1 (3.70)	6 (16.67)	
Lower thoracic vertebra (T9-12)	19 (24.68)	23 (33.82)		10 (37.04)	10 (27.78)	
Lumbar vertebra (L1-5)	40 (51.95)	18 (26.47)		12 (44.44)	6 (16.67)	
Multilevel involvement	13 (16.88)	9 (13.24)		3 (11.11)	6 (16.67)	
Fractures quantity			0.415			0.385
Single fracture	30 (38.96)	32 (47.06)		12 (44.44)	11 (30.56)	
Multiple fractures	47 (61.04)	36 (52.94)		15 (55.56)	25 (69.44)	

### Radiomics feature selection and clinical predictor analysis

**Figure 3:**
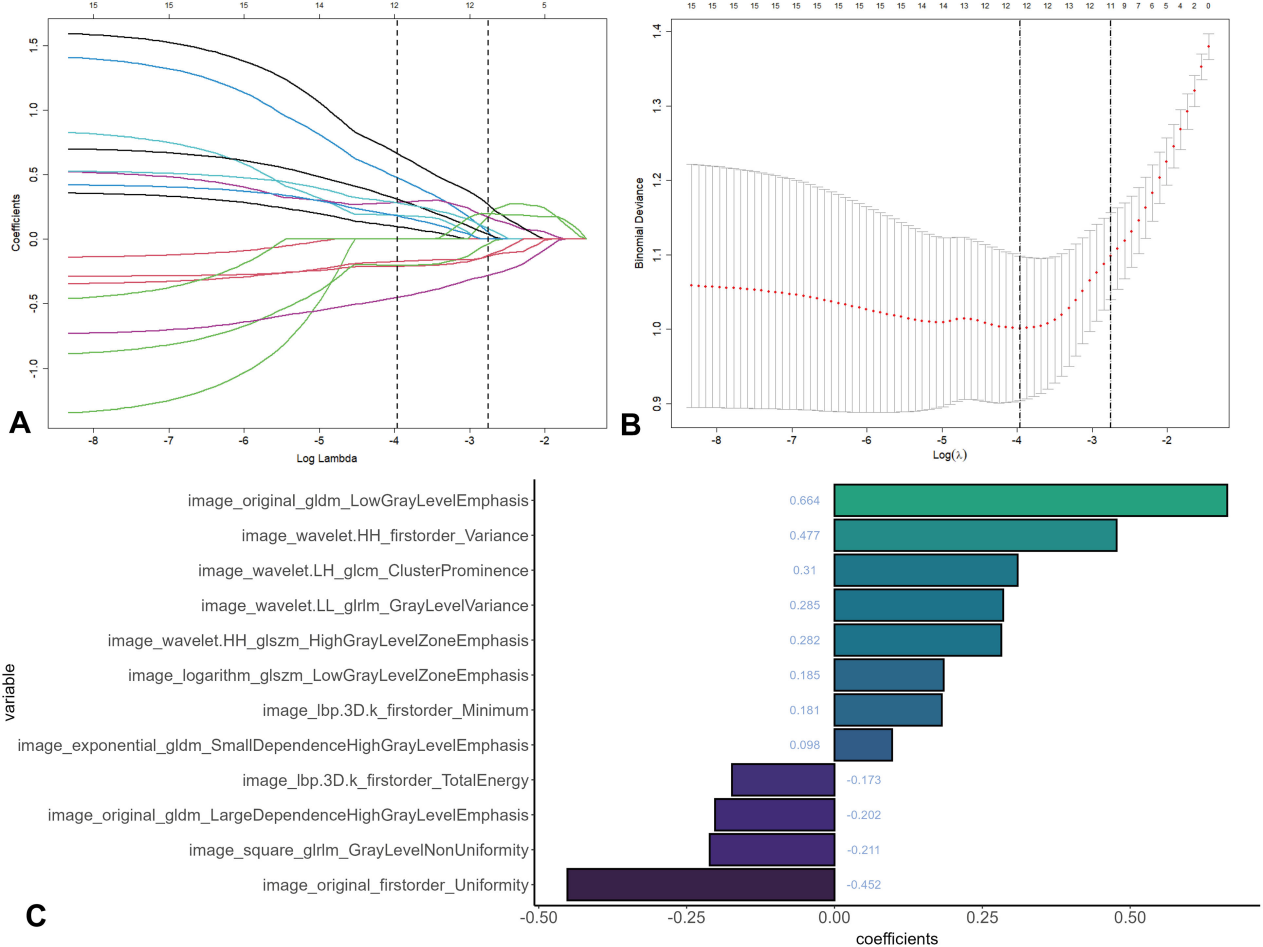
Feature selection using LASSO regression with 5-fold cross-validation. **(A)** LASSO coefficient profiles of radiomics features. **(B)** Optimal feature selection via cross-validation. **(C)** Coefficients histogram of the selected features. LASSO, least absolute shrinkage and selection operator.

A comprehensive, multi-stage feature selection process was carried out. Commencing with 874 features that met the stability criterion (ICC > 0.80), “mRMRe” package was first utilized to remove redundant features. By applying the mRMR algorithm, the feature set was reduced to 15 independent features. Subsequently, LASSO regression with 5-fold cross-validation was employed to further optimize the feature subset. As illustrated in **Figures 3A, B**, through the 1-standard error criterion, the optimal penalty parameter λ was determined to be 0.019. This led to the selection of 12 features with nonzero coefficients for the radiomics signature construction. The final feature set consisted of 4 first-order statistics, which capture basic intensity-related information such as mean, variance, and skewness of the CT image intensity distribution within the vertebral ROI, and 8 texture descriptors, which likely reflect the spatial arrangement of pixel intensities and can provide insights into the microstructural characteristics of the fractured vertebrae (**Figure 3C**). The calculation formula for the Rad-score is as follow:

Rad-score = 0.019

+ 0.664 × original_gldm_LowGrayLevelEmphasis

+ 0.477 × wavelet.HH_firstorder_Variance

- 0.452 × original_firstorder_Uniformity

+ 0.310 × wavelet.LH_glcm_ClusterProminence

+ 0.285 × wavelet.LL_glrlm_GrayLevelVariance

+ 0.282 × wavelet.HH_glszm_HighGrayLevelZoneEmphasis

- 0.211 × square_glrlm_GrayLevelNonUniformity

- 0.202 × original_gldm_LargeDependenceHighGrayLevelEmphasis

+ 0.185 × logarithm_glszm_LowGrayLevelZoneEmphasis

+ 0.181 × lbp.3D.k_firstorder_Minimum

- 0.173 × lbp.3D.k_firstorder_TotalEnergy

+ 0.098 × exponential_gldm_SmallDependenceHighGrayLevelEmphasis.

 Univariate and multivariate logistic regression analyses were performed to identify the key clinical parameters potentially related to the differentiation of benign and malignant VCFs (**Table 2**). Univariate analysis identified carbohydrate antigen 125 (CA125) and posterior vertebral involvement as statistically significant predictors (*p* < 0.05). Multivariate stepwise logistic regression further confirmed CA125 (OR = 1.02, 95% CI: 1.005-1.035, *p* = 0.008) and posterior vertebral involvement (OR = 6.231, 95% CI: 2.272-17.091, *p* < 0.001) as independent predictors.

**Table 2:** Results of univariate and multivariate logistic regression analyses.

Clinical parameters	Univariate logistics regression		Multivariate logistics regression	
Variable	OR (95%CI)	*p* value	OR (95%CI)	*p* value
Age	0.983 (0.956-1.008)	0.190		
Sex	1.68 (1.080-2.658)	0.023		
Cervical vertebra (C1-7)	1	/		
Upper thoracic vertebra (T1-4)	31302 (0-1.709)	0.987		
Middle thoracic vertebra (T5-8)	0.3 (0.013-2.936)	0.344		
Lower thoracic vertebra (T9-12)	0.242 (0.012-1.674)	0.213		
Lumbar vertebra (L1-5)	0.09 (0.005-0.610)	0.033		
Multilevel involvement	0.138 (0.007-1.050)	0.093		
Multiple fractures	1.058 (0.622-1.806)	0.832		
Wedge deformity	1	/		
Biconcave deformity	1.514 (0.655-3.568)	0.334		
Sunken	0.116 (0.006-0.646)	0.044		
Non-loss of height	0.165 (0.009-0.988)	0.099		
Neoplasm invasiveness	18122 (0-NA)	0.986		
Combined patterns	1.93 (0.444-9.909)	0.389		
Sac compression	1.136 (0.133-9.685)	0.900		
Fragment backward	1.487 (0.551-4.132)	0.434		
Retropulsion of a posterior bone fragment	0.555 (0.260-1.155)	0.120		
Posterior vertebral involvement	5.111 (2.116-13.780)	0.001	6.231 (2.272-17.091)	0.000
Paraspinal mass	0.915 (0.346-2.364)	0.855		
Spinal cord morphology	0.86 (0.446-1.653)	0.651		
Fracture line	2.296 (0.862-6.837)	0.110		
Convex posterior vertebral border	0.929 (0.458-1.874)	0.838		
LDH	1.003 (0.999-1.009)	0.192		
ALP	1.009 (1.002-1.017)	0.023		
WBC	1.03 (0.935-1.139)	0.548		
Anemia	1 (0.981-1.020)	0.986		
CA125	1.017 (1.006-1.032)	0.010	1.02 (1.005-1.035)	0.008
CEA	1.143 (0.305-4.284)	0.839		

OR, odds ratios; CI, confidence intervals; LDH, lactate dehydrogenase; ALP, alkaline phosphatase; WBC, white blood cell; CA125, carbohydrate antigen 125; CEA, carcinoembryonic antigen.

### Predictive model development and validation

**Figure 4:**
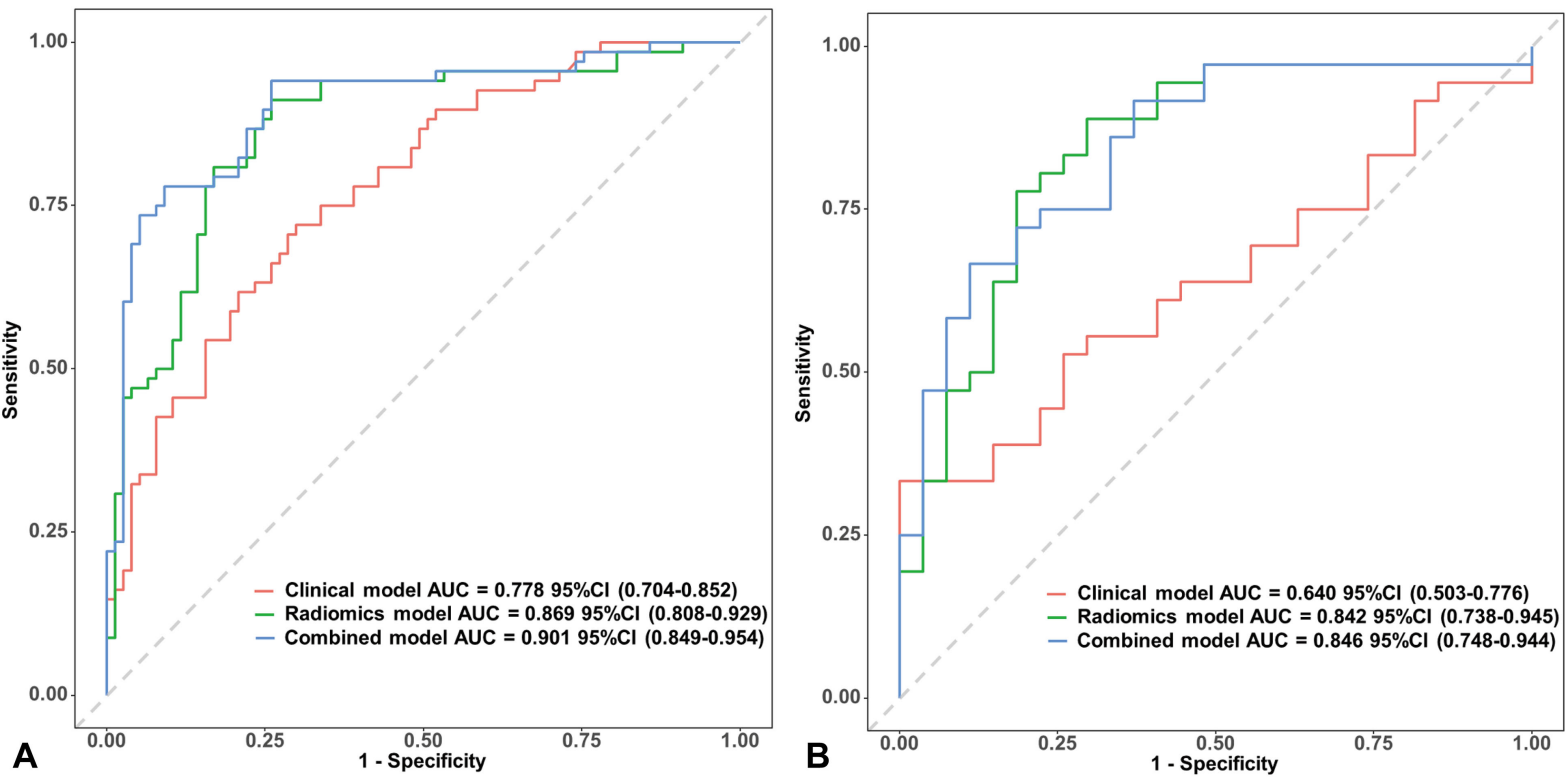
Receiver operating characteristic curves of all the models in training set **(A)** and validation set **(B)**. AUC, area under the curve; CI, confidence intervals.

Three predictive models were constructed: a radiomics model using the 12 selected radiomics features, a clinical model using the two identified clinical predictors (CA125 and posterior vertebral involvement), and a combined model integrating both Rad-score and clinical predictors as follow:


Logit (p)=−1.03448+0.78442×Rad−score+1.3997×Posterior vertebral involvement+0.02057×CA125

where *p* is the predicted probability of malignant VCFs. ROC curves for all models are shown in **Figure 4** and their differential diagnosis performance is presented in **Table 3**. The radiomics model demonstrated better performance compared to the clinical model, as reflected by higher AUC in both the training cohort (AUC: 0.869 *vs.* 0.778) and the validation cohort (AUC: 0.842 *vs.* 0.640). Notably, after integration of the clinical and imaging features, the combined model exhibited the optimal classification performance, achieving an AUC of 0.901 (95% CI: 0.849-0.954) in the training cohort and 0.846 (95% CI: 0.748-0.944) in the validation cohort. AUC differences across the three models were statistically compared using the Delong test. In both the training and validation cohorts, the combined model demonstrated statistically significant difference compared to both the radiomics model and clinical model (*p* < 0.05), but no significant difference was observed between the radiomics model and clinical model (*p* > 0.05 for both cohorts). Model performance evaluation and nomogram construction

**Table 3:** The diagnosis performance of the predictive models.

Model	AUC	ACC	SEN	SPE	PPV	NPV
Clinical model						
Training	0.778 (0.704-0.852)	0.710 (0.708-0.713)	0.721 (0.614-0.827)	0.701 (0.599-0.804)	0.681 (0.573-0.788)	0.740 (0.639-0.840)
Validation	0.640 (0.503-0.776)	0.619 (0.612-0.626)	0.333 (0.179-0.487)	1.000 (1.000-1.000)	1.000 (1.000-1.000)	0.529 (0.392-0.666)
Radiomics model						
Training	0.869 (0.808-0.929)	0.821 (0.819-0.823)	0.912 (0.844-0.979)	0.740 (0.642-0.838)	0.756 (0.663-0.849)	0.905 (0.832-0.977)
Validation	0.842 (0.738-0.945)	0.810 (0.805-0.814)	0.889 (0.786-0.992)	0.704 (0.531-0.876)	0.800 (0.676-0.924)	0.826 (0.671-0.981)
Combined model						
Training	0.901 (0.849-0.954)	0.848 (0.847-0.850)	0.779 (0.681-0.878)	0.909 (0.845-0.973)	0.883 (0.802-0.965)	0.824 (0.742-0.905)
Validation	0.846 (0.748-0.944)	0.762 (0.756-0.768)	0.667 (0.513-0.821)	0.889 (0.770-1.000)	0.889 (0.770-1.007)	0.667 (0.513-0.821)

The data in parentheses represent the 95% confidence intervals

AUC, area under the curve; ACC, accuracy; SEN, sensitivity; SPE, specificity; PPV, positive predictive value; NPV, negative predictive value.

**Figure 5:**
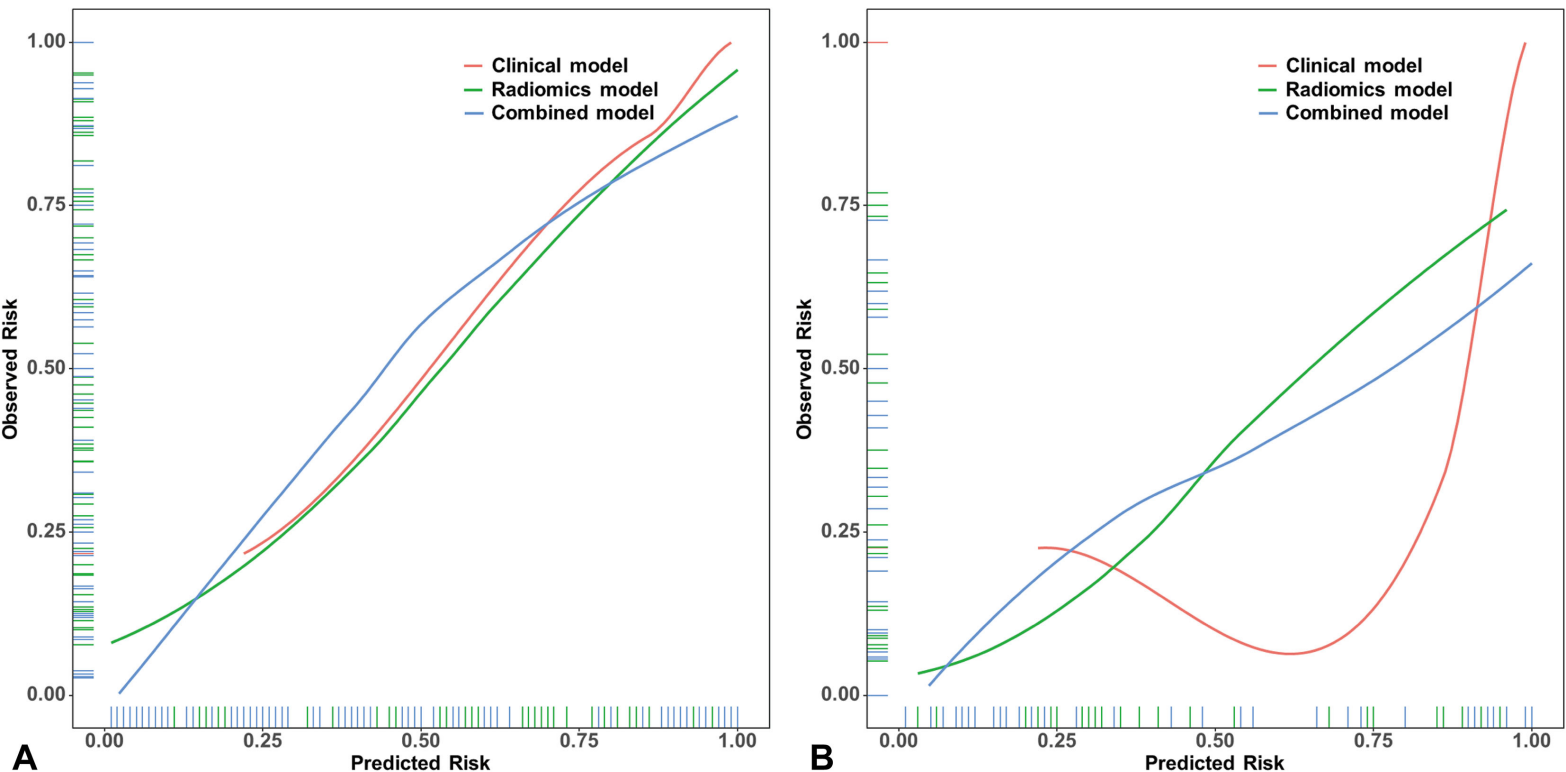
Calibration curves for malignant vertebral compression fracture prediction in training set **(A)** and validation set **(B)**.

**Figure 6:**
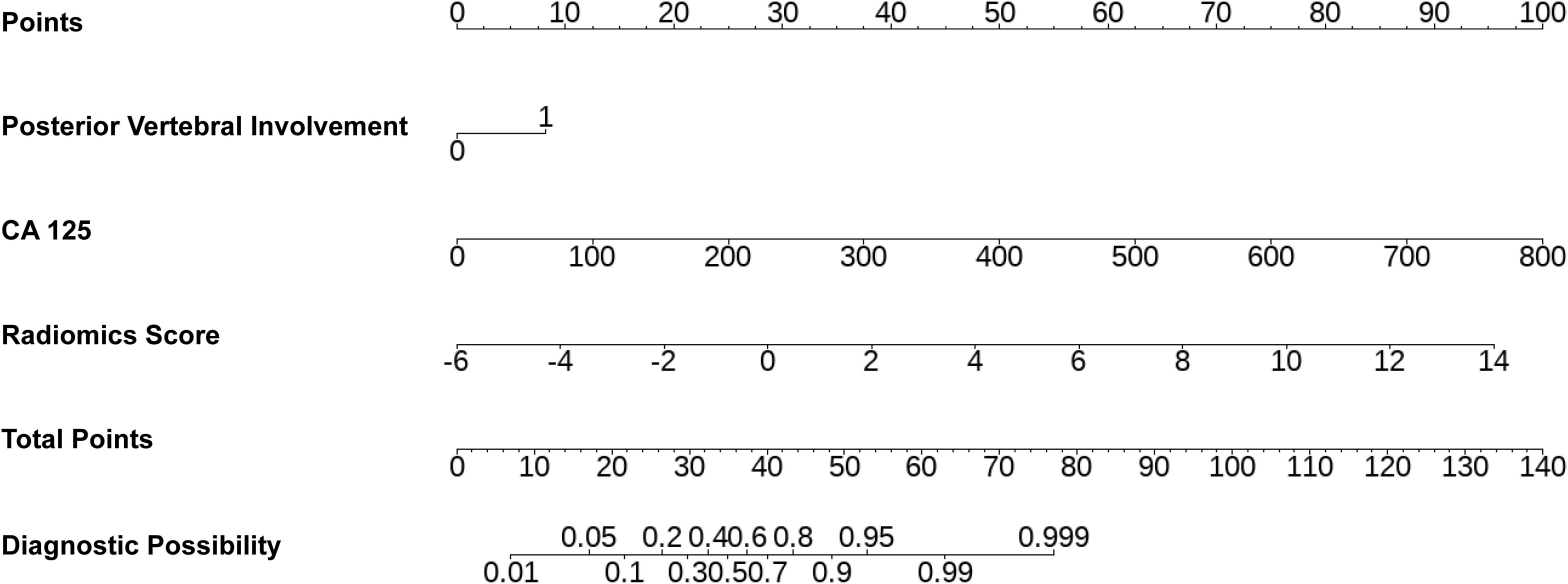
Nomogram integrating radiomics signature and clinical predictors for individualized diagnosis. CA125, carbohydrate antigen 125.

**Figure 7:**
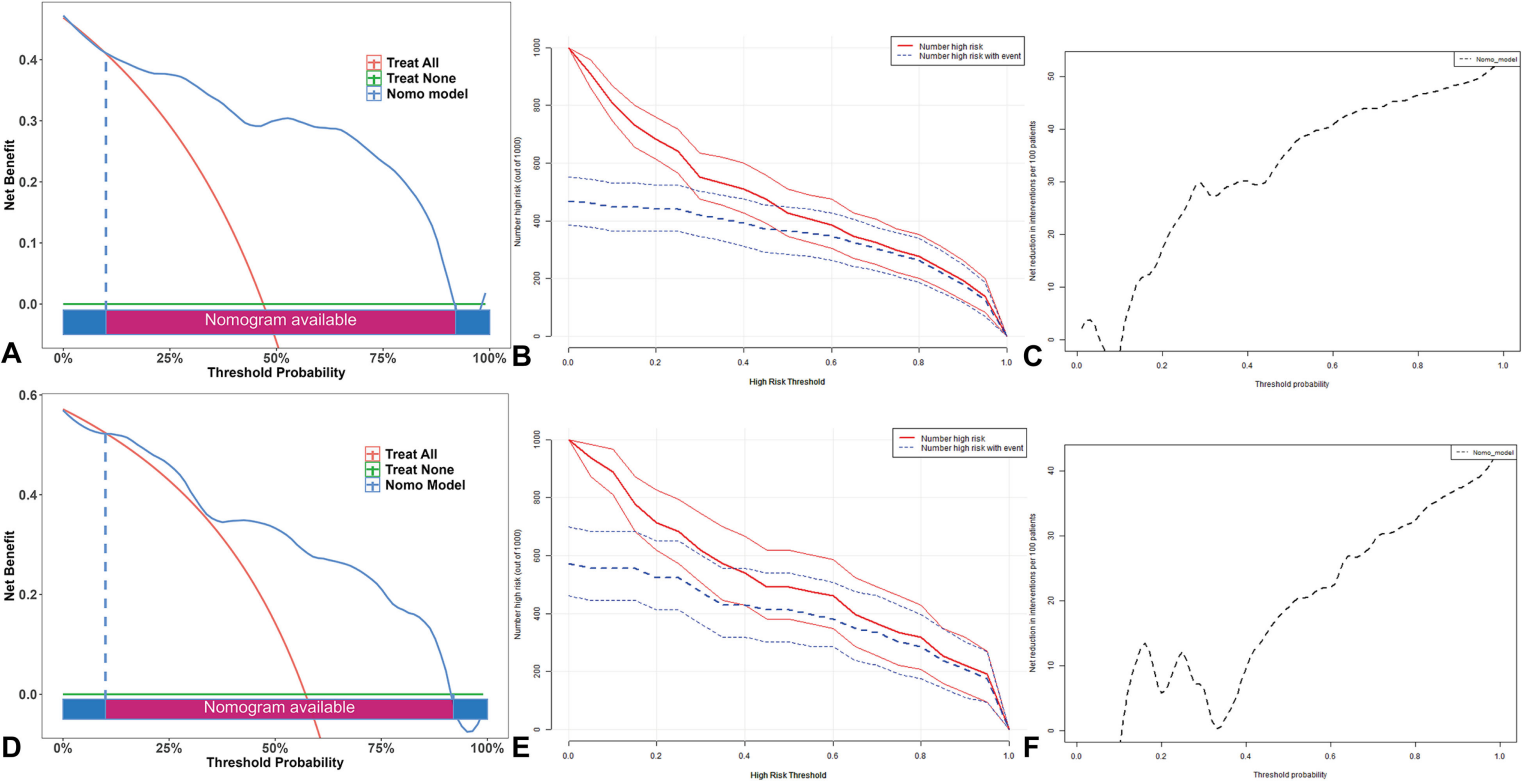
Decision curve analysis, clinical impact curves and net reduction curves of the nomogram of clinical utility in training set **(A–C)** and validation set **(D–F)**.

Calibration curves were generated to assess the agreement between predicted probabilities and observed outcomes (**Figure 5**). The combined model demonstrated favorable calibration performance in both training and validation cohorts, surpassing the radiomics and clinical models. A nomogram was constructed based on the key predictive factors from the combined model (**Figure 6**). DCA showed that in both cohorts, within the clinically actionable threshold probability range (20%-50%), the nomogram achieved a net benefit of 0.10-0.35 and outperformed the ‘Treat All’ and ‘Treat None’ extreme strategies, confirming its superior clinical value in guiding reasonable clinical decisions (**Figures 7A, D**). CIC further demonstrated that for both cohorts, as the high-risk threshold increased, the number of identified high-risk patients (solid red) and true events among these patients (dashed blue) decreased gradually (**Figures 7B, E**). The nomogram balanced the scale of high-risk populations and the proportion of true positives at practical thresholds, supporting its clinical feasibility. Finally, the upward-trending net reduction curves in both cohorts indicated that optimizing threshold probabilities enabled the nomogram to cut down unnecessary interventions per patient, highlighting its utility in mitigating overtreatment (**Figures 7C, F**). To utilize the nomogram, clinicians can assign a specific score to each predictor. For example, CA125 levels correspond to a certain number of points according to a predefined scale, while posterior vertebral involvement is scored as 1 if present and 0 if absent. The total score is then calculated by summing the individual points, enabling estimation of the probability that a VCF is malignant.

## Discussion

In this study, we developed and validated models based on CT images for differentiating benign and malignant VCFs. Our results showed that the radiomics model exhibited a higher AUC than the clinical model. Although the radiomics model did not demonstrate statistically superior discriminative ability compared to the clinical model alone (Delong test *p* > 0.05), its integration with clinical predictors significantly enhanced diagnostic performance. The combined model achieved optimal efficacy with AUC values of 0.901 in the training cohort and 0.846 in the validation cohort. This synergy establishes radiomics as a valuable supplement to clinical diagnostics in vertebral fracture assessment. Collectively, CT-derived radiomics features can serve as a reliable tool for differentiating benign and malignant VCFs, holding significant potential to assist in personalized diagnosis and treatment strategies for VCF patients.

This study identified posterior vertebral involvement and CA125 as independent predictors for differential diagnosis between benign and malignant VCFs by univariate and multivariate logistic regression analyses. The posterior vertebral elements, including the pedicles and posterior cortex, are frequently implicated in malignant lesions due to their rich trabecular bone composition and anatomical proximity to venous pathways (23, 24). Previous studies have documented that characteristics such as posterior cortical bulging and pedicle enhancement are significantly more prevalent in malignant VCFs compared to OVCFs (25), suggesting that posterior structural abnormalities may serve as imaging markers for tumor invasion. Our findings align with these observations and underscore the diagnostic significance of posterior vertebral involvement in the assessment of VCFs. CA125, a well-established tumor-associated glycoprotein (26), is elevated in various malignancies due to tumor cell proliferation and secretion, including lung, gastrointestinal, and breast cancers, as well as lymphomas (27, 28). In our study, CA125 demonstrated an odds ratio of 1.02, underscoring its strong discriminatory ability between benign and malignant cases. The findings of this study indicate that CA125 has the potential to function as a noninvasive biomarker that enhances the identification of malignant VCFs when used in conjunction with imaging features. Despite the common consideration of clinical variables such as age and history of malignancy as risk factors for vertebral metastasis (11, 18), our analysis did not find them to possess independent predictive value. Their lack of independent significance in our cohort may reflect differences in patient demographics or study design. This underscores the importance of integrating both structural and biochemical indicators into clinical decision-making models. The identification of these two key predictors also provides a rationale for subsequent radiomics modeling based on anatomical and functional characteristics.

Our findings demonstrate that CT-derived radiomics features could effectively discriminate between benign and malignant VCFs, and its diagnostic performance further enhanced through the integration of clinical variables. This further supports the utility of radiomics in capturing the underlying pathological differences between benign and malignant VCFs (14). The developed radiomics signature, comprising 4 first-order statistical features and 8 texture descriptors, effectively captures the heterogeneous characteristics of benign and malignant VCFs across two dimensions: voxel intensity distribution and spatial texture relationships. First-order features, including skewness, energy, and minimum value, quantify aspects such as the asymmetry in gray-level distribution, signal aggregation trends, and density variations within ROI. Texture features, on the other hand, reveal histological differences in lesional microarchitecture through the analysis of spatial correlations. Specifically, among all the features (**Figure 3C**), the positive regression coefficient of original_gldm_LowGrayLevelEmphasis (+ 0.664) is the largest. This finding suggests that malignant fracture regions are more likely to exhibit clustered distributions characterized by low gray levels and small dependence pixel pairs. Such distributions were particularly pronounced following wavelet decomposition. Positive associations were observed forwavelet.HH_firstorder_Variance (+ 0.477) and wavelet.LH_glcm_ClusterProminence (+ 0.310), indicating higher overall signal intensity within malignant VCFs. Previous studies have also indicated that the distribution of cells and matrix within tumor tissues can form repetitive textural patterns (29). Pathophysiologically, malignant fractures are characterized by the destruction of trabecular architecture due to tumor infiltration, which is then largely replaced by more homogeneous soft-tissue density (30). This often results in a distinct gray-level distribution that can be quantified by features like skewness and minimum value (31). Conversely, original_firstorder_Uniformity (- 0.452) had a smaller value in benign fractures compared to malignant fractures, indicating greater heterogeneity within benign fracture regions. Benign VCFs undergoing reparative processes exhibit greater attenuation heterogeneity, resulting from a mixture of fatty marrow, edematous tissue, and sclerotic bone (31). By employing multi-domain feature fusion, our study successfully captured these intrinsic differences between benign and malignant lesions, particularly in terms of gray-level distribution symmetry and textural homogeneity. This finding is consistent with previous research (11, 32). This CT radiomics-driven quantitative approach, utilizing a multidimensional feature ensemble, significantly enhances the analytical capability for discerning complex textural patterns, thereby providing valuable insights into the distinct pathological heterogeneities of benign and malignant VCFs.

Our study has several limitations. Firstly, this study is a single center retrospective analysis. Despite thorough internal validation, our homogeneous data source might limit the models’ generalizability. Secondly, we adhered rigorously to standardized protocols to minimize biases in image acquisition and feature extraction. Nonetheless, the ROI for radiomics feature extraction was delineated manually. While this approach facilitates precise targeting, it may still result in inter-observer variability, notwithstanding our standardization efforts. Therefore, multicenter collaborations and larger sample sizes are desirable for future studies.

## Conclusion

In conclusion, the CT radiomics-clinical integrated model developed in this study exhibited a robust diagnostic performance in distinguishing between benign and malignant VCFs. This model underscores the significance of radiomics as a vital complement to traditional imaging diagnostics, offering clinicians an objective and quantifiable decision-support tool for patient management.

## Data Availability

The original contributions presented in the study are included in the article/supplementary material. Further inquiries can be directed to the corresponding authors.
