# Correction: Differential diagnosis of benign and malignant vertebral compression fractures based on CT radiomics model

**DOI:** 10.3389/fonc.2026.1784208

**Published:** 2026-01-22

**Authors:** Xinrui Liu, Song Chen, Yifan Wang, Jiashi Cao, Zhuangfei Niu, Yuxian Jin, Xingdan Pan, Zhengwei Zhang, Tielong Liu, Wei Liang, Panfeng Yu, Weiwei Zou

**Affiliations:** 1 School of Health Science and Engineering, University of Shanghai for Science and Technology, Shanghai, China; 2 Shanghai Changzheng Hospital Department of Radiology, Shanghai, China; 3 Shanghai Baoshan District Wusong Central Hospital (Zhongshan Hospital Wusong Branch, Fudan University), Shanghai, China; 4 Navy Medical Center, The Navy Medical University, Shanghai, China; 5 Shanghai Changzheng Hospital Department of Orthopedic Oncology, Shanghai, China; 6 Department of Pathology, Shanghai Changzheng Hospital, Shanghai, China; 7 Shanghai 411 Hospital, Affiliated Hospital of Shanghai University, Shanghai, China; 8 Department of Orthopedic Oncology, Peking University People’s Hospital, Beijing, China

**Keywords:** clinical predictors, computed tomography (CT), machine learning, radiomics, vertebral compression fractures (VCF)

Affiliation “Navy Medical Center, The Navy Medical University, Shanghai, China” was omitted for author Yifan Wang. This affiliation has now been added for author Yifan Wang.

Author Yifan Wang was erroneously assigned to affiliation “Department of Pathology, Shanghai Chang-zheng Hospital, Shanghai, China”. This affiliation has now been removed for author Yifan Wang.

The funder “Shanghai Medical Field Rising Stars Young Medical Talents Cultivation Program”, to Weiwei Zou was erroneously omitted, the correct funding statement reads:

“The author(s) declared that financial support was received for this work and/or its publication. The work was supported by the National Natural Science Foundation of China (Grant No. 82372609), Shanghai University of Science and Technology Key Support Program for Interdisciplinary Research in Medicine and Engineering (Grant No. slg-zww) and Shanghai Medical Field Rising Stars Young Medical Talents Cultivation Program (Grant No. YYXXZWW).”

The original version of this article has been updated.

